# Serial Examination of Platelet Function Tests Might Predict Prognosis of Patients with Acute Ischemic Stroke—A Cohort Study

**DOI:** 10.3390/diagnostics14242848

**Published:** 2024-12-18

**Authors:** Hsin-Ju Ho, Li-Ching Wu, Yu-Wei Chen

**Affiliations:** 1 Department of Biomedical Science and Engineering, Program in Systems Biology and Bioinformatics, National Central University, Taoyuan 320317, Taiwan; 104886001@cc.ncu.edu.tw (H.-J.H.); richardw@ncu.edu.tw (L.-C.W.); 2 Department of Neurology, National Taiwan University Hospital, Taipei 100225, Taiwan; 3 Department of Neurology, Landseed International Hospital, Taoyuan 324609, Taiwan; 4 Center for General Education, National Central University, Taoyuan 320317, Taiwan

**Keywords:** platelet function analyzer 100, ischemic stroke, modified Rankin Scale, clinical outcome, prognosis

## Abstract

Background: This study investigated whether point-of-care platelet function measurements could predict favorable outcomes in patients with acute ischemic stroke (AIS). Antiplatelet agents, such as aspirin, are known to reduce the risk of recurrent stroke by 20–30%. However, identifying nonresponders to therapy remains a clinical challenge. The study aimed to assess the prognostic value of serial Platelet Function Analyzer (PFA)-100 measurements and hematological ratios in AIS patients. Methods: A prospective cohort study was conducted on 212 AIS patients in Taiwan. Platelet function was assessed at baseline, week 2, and week 4 using PFA-100. The primary outcome was functional recovery, defined by a modified Rankin Scale (mRS) score of 0–3, at 1-month and 1-year. Subgroup analyses compared outcomes between pre- and post-aspirin administrations. Statistical analyses examined the association between changes in platelet function and clinical outcomes. Results: Difference in collagen and epinephrine (CEPI) measurements between baseline and week 2 was associated with favorable mRS scores (*p* < 0.001). A difference in CEPI closure time greater than 99 seconds was most predictive of a favorable outcome with an adjusted odds ratio of 11.859 (95% CI 2.318–60.669) at 1-month follow-up. Subgroup analyses revealed predictive value in pre-aspirin measurements at 1-month follow-up (*p* = 0.007). Conclusions: Serial PFA-100 measurements and hematological biomarkers, specifically changes in on-treatment CEPI closure times, may help predict favorable clinical outcome in AIS patients. These findings suggest that dynamic platelet function assessment could play a role in optimizing antiplatelet therapy in AIS management.

## 1. Introduction

Antiplatelet agents, predominantly aspirin, can reduce the relative risk of recurrent stroke in ischemic stroke patients by approximately 11–22% [1,2,3]. However, identifying clinical nonresponders to aspirin therapy prior to the occurrence of subsequent vascular events is challenging. Furthermore, long-term use of antiplatelet medications is associated with an increased risk of hemorrhage [4]. Increased levels of biomarkers of platelet activation have been reported in the acute stage of cerebral infarction [5]. The associated changes in biomarkers on antiplatelet agents have been investigated by several laboratory examinations to determine the platelet reactivity of patients undergoing antiplatelet treatment. Several platelet function tests have been developed to support clinical judgment in the choice and adjustment of antiplatelet therapy for secondary stroke prevention [3]. One platelet function test, the Platelet Function Analyzer-100 (PFA-100), has the advantage of bypassing the platelet preparation stage and closely mimics natural physiological conditions. It has been shown to be valuable in patients with acute coronary syndrome [6], with results that correlate with cardiovascular outcomes [7]. The PFA-200 analyzer, introduced in the mid-2010s, as the intended successor to the PFA-100, is predominantly available in European countries and Australia [2]. Although PFA-200 measurements in the chronic stage might not correlate with prognosis [8], it has shown sensitive to aspirin intake in stroke patients [9]. Additionally, peripheral blood cell count ratios, as inflammatory indicators are reported be associated with clinical outcome of acute stroke [10,11,12,13]. The aim of this study was to investigate the clinical applications of serial PFA-100 measurements of platelet reactivity and hematological ratios in predicting the prognosis of acute ischemic stroke (AIS).

## 2. Materials and Methods

### 2.1. Overview

We conducted a prospective, single-center, observational cohort study on consecutive AIS participants aged 18 years or older admitted to the Landseed International Hospital in Taiwan from June 2009 to November 2010. All participants provided informed consent before enrolling in the study. The study was conducted in accordance with the Declaration of Helsinki, and the protocol was approved by the Institutional Review Board (IRB) of Landseed Hospital (LS-IRB-09-14).

### 2.2. Participants

The participants were diagnosed with acute cerebral infarction, which was verified within 10 days of symptom onset through comprehensive neuroimaging examinations, as well as pre-specified clinical evaluations by qualified neurologists. During the recruitment period, a total of 375 participants diagnosed with stroke were enrolled, along with 112 healthy volunteers without any history of stroke. The exclusion criteria included prior use of antiplatelet agents before the index events; no prescription of antiplatelet agents between measurements; diagnosis of transient ischemic attack (TIA) by neurologists; initial hematocrit < 30% or platelet counts < 150,000/L; intake of nonsteroid analgesic agents between measurements; and lack of informed consent for follow-up. Healthy volunteers without any hemostatic abnormalities and who had not taken any medication prior to enrollment served as health control group.

The National Institute of Health Stroke Scale (NIHSS) score was used to measure stroke severity. To assess the severity of neurological deficits, participants with stroke were divided into two subgroups: mild neurological deficit (0–8 points on the NIHSS) and moderate to severe neurological deficits (9 points or more on the NIHSS).

The primary outcome was the relationship between 1-month (1-M) and 1-year (1-Y) favorable functional outcome, defined as a modified Rankin Scale (mRS) score of 0 to 3, and the PFA-100 measurement taken at day 1 (baseline), week 2 and week 4 after onset, as well as their difference.

### 2.3. Clinical Measurement

All participants with acute cerebral infarction received antiplatelet medication, consisting of aspirin alone, or in combination with clopidogrel or dipyridamole, immediately upon a definitive diagnosis. During the study period, aspirin 100-300 mg was given as the loading dose, followed by 100 mg daily in accordance with the treatment guidelines. PFA-100 measurements were performed immediately after the study protocol was explained and informed consent was signed by the participants or their legal representatives, aligned with Landseed International Hospital’s IRB requirements. Therefore, PFA-100 measurements could be conducted either before or after aspirin administration following informed consent procedures. For subsequent follow-up visits at weeks 2 and 4, PFA-100 measurements were conducted in the hospital or outpatient department if they were discharged. Medication compliance was confirmed in inpatients by the attending nurses, and verified through personal interviews throughout the study period in outpatient department visits. During hospitalization and after discharge, relevant clinical and laboratory data were prospectively recorded following predetermined protocols to ensure consistency and accuracy.

To investigate the influence of aspirin on PFA-100 measures, we divided the participants into subgroups based on their baseline measurement taken before or after receiving antiplatelet therapy. Subgroup analyses were then conducted to compare results and outcomes between the pre-aspirin (Pre-ASA) and post-aspirin (Post-ASA) subgroups.

Acute stroke therapies often use the mRS to assess functional outcomes and activity limitations. The functional status was evaluated at two different time points: 1-M (early prognosis) and 1-Y (late prognosis) after stroke onset. The participants were followed-up mainly primarily through personal interview or telephone for a period of 1-Y.

For the functional status of participants, two subgroups were identified: those with a favorable prognosis (0–3 points on the mRS) and those with an unfavorable prognosis (4–6 points on the mRS).

A group of participants without a previous history of cerebrovascular disease or coronary artery disease was recruited to simulate the platelet function results of general population, as measured by PFA-100.

### 2.4. Blood Collection and Platelet Function Analyzer-100 Closure Time Determination

A complete blood cell count was obtained at the time of emergency room or initial hospital admission. The count included total white blood cell, platelets, neutrophil, lymphocyte, monocyte, eosinophil and basophil counts. Three inflammatory markers were calculated from these measurements: neutrophil-to-lymphocyte ratio (NLR), platelet-to-lymphocyte ratio (PLR) and lymphocyte-to-monocyte ratio (LMR) [10].

Blood samples for PFA-100 were collected from participants upon initial presentation of acute ischemic stroke (day 1, baseline), with subsequent measurements taken at two and four weeks after the first assessment. The samples were left at room temperature for 30 min to stabilize and were analyzed within 3 h of collected.

To measure platelet function, the PFA-100 (Dade Behring Inc. 1717 Deerfield Road, Deerfield, IL, USA) was used to determine the closure time (CT) when the blood sample with 106 mmole/L (3.2%) sodium citrate flowed through cartridges with membranes coated by collagen and epinephrine (CEPI) or collagen and adenosine diphosphate (CADP). During the test, a blood sample is passed through a capillary at high shear rates, causing the agents to contact with CEPI or CADP. This interaction causes platelets to adhere and aggregate until a platelet plug completely clogs the aperture. The time needed to stop the blood flow and to obtain the occlusion is defined as the CT. The maximal value for the CT is 300 seconds (s), as provider by the manufacturer [14]. If the CT exceeds 300 s, it is reported as nonclosure. Our laboratory’s normal ranges for the collagen and epinephrine closure time (CEPI-CT) are 82 to 150 s, and for collagen and adenosine diphosphate closure time (CADP-CT) are 62 to 100 s, as provided by the manufacturer [2,15]. Both CEPI-CT and CADP-CT values of 300 s are arbitrarily assigned for conservative statistical comparisons.

Medication taken between measurements, such as antiplatelet agents and nonsteroidal anti-inflammatory drugs, was also documented. The study was designed to perform PFA-100 tests at three serial measures at baseline, week 2 and 4, respectively. Previous studies indicate that the CADP cartridge lacks sensitivity to aspirin therapy [16,17,18]. Therefore, further statistical analysis emphasized the values of CEPI-CT at three time points and their differences, given its greater sensitivity to aspirin treatment, which is the mainstay of antiplatelet agents in secondary prevention in AIS.

### 2.5. Statistics

We compared the baseline characteristics and changes in mRS scores between groups categorized by outcome (favorable vs. unfavorable) at the 1-M and 1-Y follow-ups. Comparisons of normally distributed continuous data were performed using parametric *t*-tests, while nonparametric Mann–Whitney *U* tests were used for non-normally distributed continuous variables. Categorical data were analyzed using chi-squared or Fisher’s exact tests. Spearman’s rank test was used to evaluate the correlations between the variables. The quartiles for changes in CEPI from baseline to week 2 assessment were calculated by outcome at 1-M and 1-Y follow-up. Favorable and unfavorable outcome participants were compared across all covariates using logistic regression analysis. We performed univariate logistic regression analysis for all potential variables. Variables with a *p*-value less than 0.05 in univariate analysis were subsequently included in the multivariate logistic regression model to adjust for confounding factors associated with functional outcome [12]. A series of multivariable-adjusted logistic regression models were used to examine the associations between groups categorized by outcome (favorable vs. unfavorable) [19]. Prespecified stratified analyses were conducted by mRS (0–3 and 4–6) while controlling for the variable in the models. We repeated the same analysis for the outcome at the 1-Y follow-up.

A two-sided *p* < 0.05 was considered significant. SAS software (version 9.4; SAS Institute, Inc., 100 SAS Campus Drive, Cary, NC, USA) was used to perform all statistical analyses.

## 3. Results

### 3.1. Baseline Characteristics and Participants Overview

**Figure 1:**
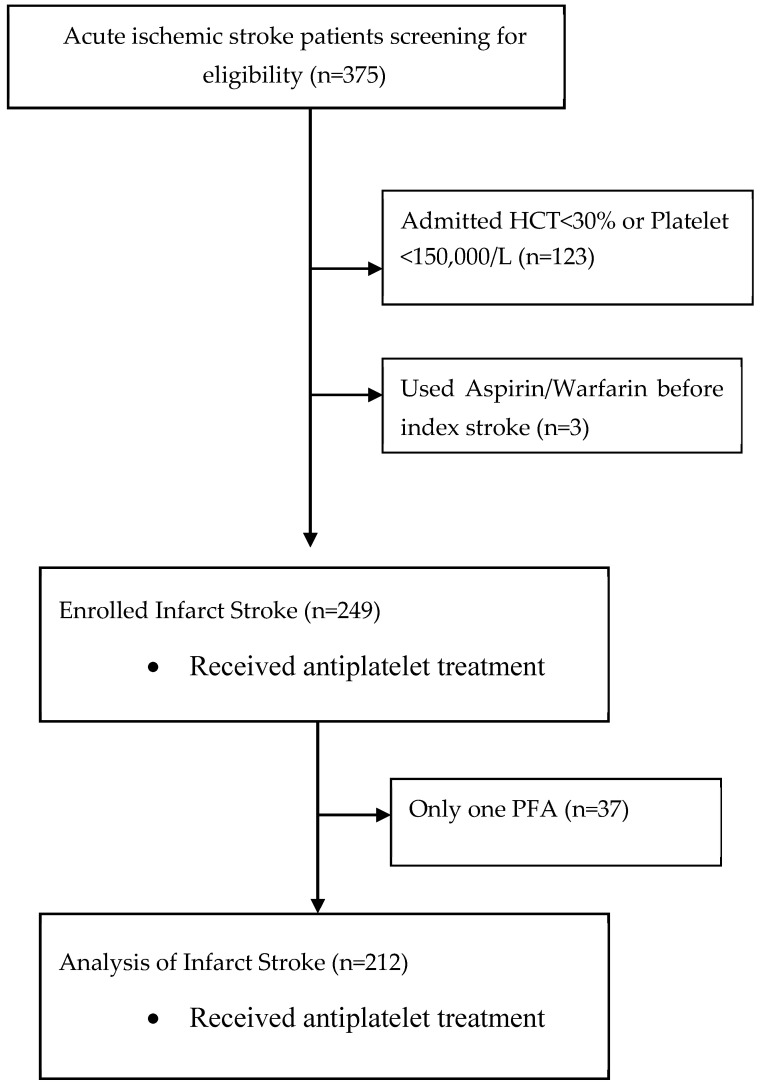
Study flow chart diagram showing the number of participants screened, excluded, and analyzed. HCT: hematocrit; PFA: Platelet Function Assay.

**Figure 2:**
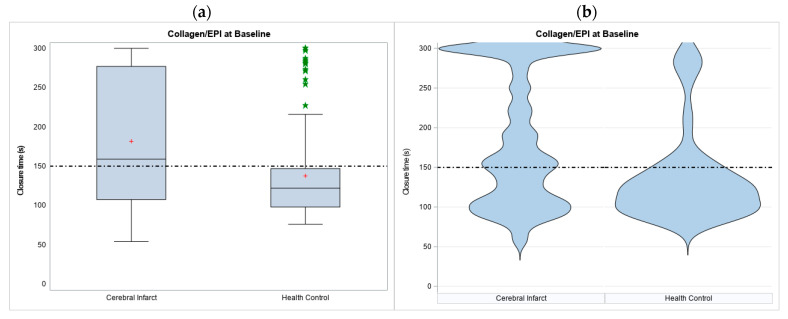
Study of participants’ CEPI of PFA assessment at baseline (Day 1). (**a**) The boxes represent the interquartile range (IQR) with median line inside. Whiskers extend to the most extreme data points within 1.5 × IQR. Outliers beyond the whiskers are shown as green asterisks (*). The mean values are indicated by red crosses (+). The horizontal dashed line at 150 serves as a reference value, indicating the upper limit of normal range for CEPI-CT. (**b**) violin plot for the distribution of the relative patient numbers distributed in CEPI-CT seconds. s, seconds; PFA, platelet function analysis; CEPI: collagen and epinephrine.

Among 375 acute ischemic stroke participants enrolled in the study, we excluded participants with prior use of antiplatelet or warfarin before the index events (*n* = 3), initial hematocrit < 30% or platelet counts < 150,000/L (*n* = 123) and those with missing data on week 2 and week 4 assessment (*n* = 37) (Figure 1). A total of 212 infarct participants were included in the analysis after applying the exclusion criteria. The baseline characteristics of the study participants are presented in Table 1 and Table 2. Patients with acute cerebral infarct were with ages of 65.04 ± 14.42 years, male predominance (63.68%), prevalent vascular risk factors, including hypertension (75%), dyslipidemia (48.11%) and diabetes mellitus (37.74%) than participants in the health control group. The overall baseline CEPI-CT was also significantly longer in infarct group than health control group (181.71 ± 81.50 vs. 137.63 ± 57.50, *p* < 0.001) (Table 1, Figure 2). Only 39.6% of infarct patients had CEPI-CT within 82–150 s reference ranges by manufacturer, compared to 73.2% in the health control population. The Pre-ASA subgroup (*n* = 86) had baseline CEPI-CT of 141.0 ± 68.65 s and Post-ASA subgroup had much longer 209.82 ± 78.29 s (*p* < 0.0001).

### 3.2. Outcome

At 1-M follow-up, 158 (74.53%) achieved a favorable outcome, defined as a mRS score of 0 to 3, who were more likely to be male (70.25%), younger (62.27 ± 13.59 years). They had a significant lower prevalence of previous cerebrovascular accidents (15.19 vs. 29.63%, *p* = 0.019) and insignificantly less hypertension history (72.78 vs. 81.48%, *p* = 0.203) compared to those with unfavorable outcomes. Furthermore, they had lower initial NIHSS scores (3.32 ± 3.38), and higher Barthel index scores (94.57 ± 13.44), reflecting better performance in activities of daily living (Table 2). High baseline hemoglobin levels were significantly associated with 1-M and 1-Y favorable outcomes, and lower baseline PLR was associated with 1-M favorable outcome (Table 2).

**Figure 3:**
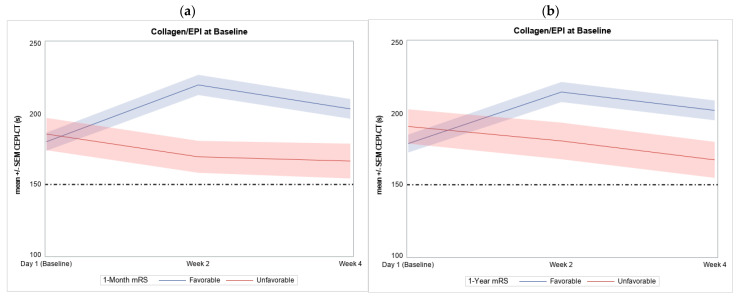
Comparison of the CEPI-CT by time points in participants with favorable and unfavorable outcome at (**a**) 1-month and (**b**) 1-year follow-up. The horizontal dashed line at 150 serves as the upper limit of normal reference ranges for CEPI-CT. s, seconds; SEM, standard error of the mean; CEPI-CT, collagen and epinephrine-closure time; mRS, modified Rankin Scale.

Among participants with favorable and unfavorable outcomes at 1-M follow-up, baseline CEPI-CT measurement did not show a significant difference (180.32 ± 80.76 vs. 185.80 ± 84.26, *p* = 0.671) (Table 3). However, significant differences were observed at week 2 and week 4 (*p* = 0.0003 and *p* = 0.012, respectively) (Table 3, Figure 3a). Similarly, at 1-Y follow-up, significant differences in CEPI-CT were recognized at week 2 and 4 between favorable and unfavorable outcome subgroups (Table 3, Figure 3b).

**Figure 4:**
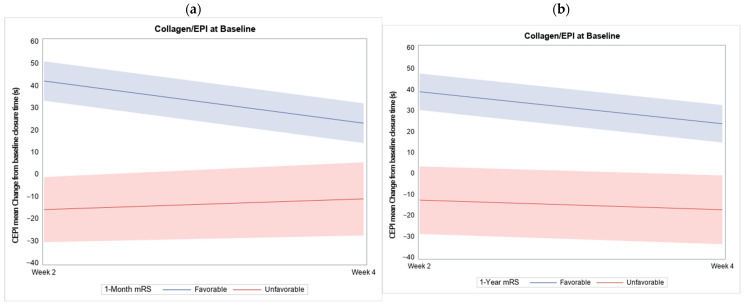
Comparison of the CEPI-CT by changes from baseline in participants with favorable and unfavorable outcome at (**a**) 1-month and (**b**) 1-year follow-up. s, seconds; CEPI-CT, collagen and epinephrine-closure time; mRS, modified Rankin Scale.

At 1-Y follow-up, 164 (77.36%) achieved a favorable outcome. Comparison of participant characteristics at 1-Y follow-up outcome revealed similar results to those observed at 1-M, except for a higher proportion of smokers. A significant prolongation in CEPI-CT from baseline to week 2 measurement was associated with a favorable outcome (42.12 ± 101.23 vs. −15.91 ± 100.69, *p* = 0.0009) (Table 3, Figure 4a).

Additionally, a significant difference in CEPI-CT between baseline and week 4 was found among participants with favorable outcomes compared to those with unfavorable outcomes at 1-Y follow-up (23.71 ± 105.38 vs. −17.31 ± 98.35, *p* = 0.036) (Table 3, Figure 4b). We demonstrated that the changes in CEPI between baseline and week 2 were correlated with inflammatory markers (NLR, PLR, and LMR) compared to the changes between week 2 and week 4. This correlation was particularly significant with PLR (*p* = 0.049), but not significant with NLR and LMR (*p* = 0.074 and *p* = 0.063, respectively).

Among the Pre-ASA subgroup, those with favorable and unfavorable outcomes either at 1-M or 1-Y had no significant differences in baseline CEPI-CT (1-M, 138.7 ± 67.85 vs. 149.3 ± 72.69, *p* = 0.555; 1-Y, 136.1 ± 66.54 vs. 159.6 ± 75.19, *p* = 0.199, respectively) (Supplementary Table S1). Similarly, the comparison neither showed significant difference in analyzing the Post-ASA subgroup (Supplementary Table S2).

We demonstrated the difference of serial PFA-100 measurements was associated with favorable outcomes in the study. The crude odds ratio (OR) at 1-M follow-up was 1.006 (95% confidence interval, [CI], 1.002–1.009, *p* = 0.002), and at 1-Y was 1.005 (95% CI, 1.001–1.008, *p* = 0.006) (model 1 in Table 4). This association remained significant in model 2, adjusted for age, sex and model 3, adjusted for age, sex, dichotomized NIHSS scores by 8. The area under the curve (AUC) for model 3 in predicting 1-M favorable outcomes was 0.91. We further adjusted for previously identified significant factors (previous CVA, NLR, and PLR) in model 4, achieving an AUC of 0.93. However, no association had been found between CEPI-CT changes from baseline to week 4 measurement and the 1-M favorable outcome in model 4 (adjusted OR (aOR) = 1.004, 95% CI, 1.00–1.008; *p* = 0.083; Table 4). We also found insignificant association between CEPI changes from week 2 to week 4 with the 1-M and 1-Y favorable outcome in this adjusted model.

In our study, we identified quartile cutoff values of −23, 21, and 99 for the change in CEPI-CT from baseline to week 2. Participants with CEPI-CT changes greater than 99 s in the upper quartile, exhibited a significantly higher adjusted OR of 11.859 (95% CI, 2.318–60.669; *p* = 0.003) for favorable outcome compared to those in the lower quartile, after adjusting for age, sex, initial subgroup NIHSS scores, previous CVA, NLR and PLR. This prognostic model had high discriminative power, as shown in Table 4 (AUC = 0.93; 95% CI, 0.88–0.97; *p* = 0.005 in 1-M). The analysis results may demonstrate the dose-response effects between CEPI-CT prolongation and clinical outcomes, as the aORs for the fourth quartile (Q4) group were significantly better than reference (Q1) group (*p* = 0.003 in 1-M and *p* = 0.004 in 1-Y). In contrast, the statistical analysis for the second (Q2) and third (Q3) quartile groups showed either insignificant or inconsistent results compared to Q1 (Table 4). The Pre-ASA subgroup, CEPI-CT significantly predictive 1-M favorable outcome across all models (model 4: OR = 1.012; 95% CI, 1.003–1.021; *p* = 0.007), while Post-ASA showed insignificant associations (Supplementary Table S3). However, the Post-ASA subgroup demonstrated significant associations between CEPI-CT changes from baseline to week 2 measurement and 1-Y favorable outcomes in models 1, 3, and 4 (model 4: OR = 1.010; 95% CI, 1.002–1.019; *p* = 0.021).

## 4. Discussion

Our study demonstrated that the individual difference of platelet functions between onset time of AIS and 2 weeks later with aspirin treatment was significantly associated with a favorable outcome at 1-M and 1-Y, whereas the one-time cutoff measure at 2 weeks after onset with aspirin was not. The measures were performed by closure time of CEPI of PFA-100 and the favorable outcome was defined by mRS 0–3.

There is wide variation using laboratory results to define “resistance” to aspirin or other antiplatelet agents for adjusting the dosage or regimen to improve clinical outcomes due to lack of well-accepted definition and standardized tests [20], and presence of individual difference to antiplatelet agents [3]. Even for a particular examination modality, the generalized clinical application has been difficult due to various reference values from healthy donors in different laboratories [2]. Besides, one could question if we could apply the reference values in patients for secondary prevention for vascular diseases since long-term aspirin use could activate alternate pathways of platelet aggregation [21]. Our study demonstrated that AIS participants had a much wider distribution and longer CEPI-CT than health control, with 28.3% having a CEPI-CT longer than 250 s (vs. 10.7% in healthy control) and some up to the upper limits of 300 s, consistence with findings in other studies [14,16,22]. However, further subgroup analysis revealed that the wide variation in CEPI-CT during the acute stages might probably influenced by whether the measures were taken before or after aspirin administration as part of emergent treatment. The baseline CEPI-CT values in the Pre-ASA subgroup were close to health control group (*p* = 0.708), but were significantly shorter than those in the Post-ASA subgroup.

We demonstrated the differences of CEPI-CT between baseline and week 2 were significantly associated with 1-M and 1-Y favorable functional recovery in our study in the adjusted models, but using either CEPI-CT 150 or 193 s as cut-off values were not. Compliance to aspirin was almost ensured during hospitalization, but less after discharge. This could possibly explain the better correlation of second measurement performed within two weeks after the first assessment. The 14-day time interval between measurements was set considering 7-day life span of platelets.

Our findings are in agreement with previous studies focusing on participants with coronary artery disease (CAD) [23,24,25,26]. In the subgroup analysis of our study, the CEPI-CT difference in Pre-ASA group was significantly associated with both 1-M and 1-Y favorable outcomes, whereas in Post-ASA group, it was significantly association only with 1-Y favorable outcomes. A previous study with serial measures of platelet function tests in AIS at 3 h and 5 days after taking aspirin found that persistent high platelet activity at both measures was significantly associated with an increased composite vascular event, including stroke, myocardial infarct, and vascular death within a year [27].

Previous studies did not demonstrate the consistent association between the platelet function tests and clinical outcomes [28,29], resulting in no consensus about the application of platelet function tests in antiplatelet uses after AIS [30]. It could be possibly explained for this inconsistency.

First, the correlations among values of various diagnostic modalities and clinical outcomes have not been well established. Light transmission aggregometry (LTA) is recognized as the gold standard for the evaluating platelet aggregation [31,32], but other methods, such as impedance aggregometry [33], thromboelastography (TEG) and PFA are also utilized with various advantages [31,34]. PFA-100 has been proposed for screening von Willbrand disease, evaluating effect of antiplatelets, either for aspirin [2], or additional function inhibition of dipyridamole [35,36]. The device is simple to use, less time consuming, requires only a small volume of blood [37]. PFA-100 results are proven to be correlated with those from impedance aggregometry and LTA in CAD [38] and secondary stroke prevention [35], but other studies demonstrated the results of different tests are not interchangeable [29,39].

Second, the reference values for specific examination differ among laboratories, limiting their clinical application. Taking PFA for example, it could be due to non- standardized laboratory methods (e.g., 3.2% vs. 3.8 citrate), concomitant food and supplements, donor population sizes, timing of sampling [31,34] and raise doubts about universal utilization of normal reference ranges (NRR) of specific laboratory, such as 82–150 s CEPI-CT using 3.2% citrate by the manufacture [2,15,40]. A recent large network evaluation recommended higher CEPI-CT 90–160 s and CADP-CT 70–124 s, respectively [41]. Aspirin resistance, defined by platelet function in the laboratory, could range from 31.5% (NRR of 94–193 s for CEPI-CT) to 13.4% using 128 s as its own reference range [28]. In our reference donor population of 112 people, with a mean age of 51.8 years, we found CADP-CT to be 70–101 and CEPI-CT to be 98–147 s, close to the manufacture’s reference. It is why the study group decided to use the manufacture’s reference in our study analysis.

Third, patients with acute ischemic arterial diseases might have different distribution of platelet function tests results compared to the normal population. Some studies support the role of platelet function tests in predicting severity of myocardial infarction [35], recurrent ischemic stroke in 90 days [42] and complication after peripheral angioplasty [43]. However, using such tests to adjust antiplatelet therapy did not improve the outcome of acute coronary syndrome [44]. This could be possibly due to the heterogeneity of various vascular diseases and of clinical scenario reflected by the values of different results of platelet function tests at onset before or after treatment. Our subgroup analysis findings suggest that the heterogeneity of PFA-100 values in the acute stroke patients could be reduced by measuring platelet function before aspirin treatment and analyses based on the pre-treatment measures might be better associated with clinical outcomes in the follow-up.

PFA-100 measurements demonstrated the highest sensitivity in predicting aspirin pretreatment [9]. Our study demonstrated that the reduced platelet activities in serial measures by PFA-100 with aspirin treatment in vivo were associated with early and late clinical favorable outcomes, particularly when it was measured before treatment. The results were consistent with previous findings that patients on aspirin treatment for more than 5 days demonstrated significantly longer CEPI-CT values in those without recurrence of ischemic stroke compared to those with recurrence (267 vs. 102.5 s) during the 13 months [28]. It might explain the significant correlation between difference in Post-ASA subgroup during the acute stage and 1-Y outcome, as these measurements in Post-ASA subgroups may not yet reflected the optimal effect of aspirin. The lack of CEPI-CT or CADP-CT prolongation in the serial measurements may indicate high on-treatment platelet activity and clinical nonresponse to aspirin [45,46].

Our study demonstrated that the difference in CEPI serial measurement might be correlated with hemoglobin, blood cell ratios, including NLR, PLR and LMR, particularly PLR. These biomarkers have been used to quantify the inflammatory and immune responses that precipitate neurotoxic effects during thromboembolic events at onset [10]. Decreased blood lymphocytes due to lymphocyte apoptosis and lower counts have been associated with worse outcomes [47]. The correlation between CEPI difference and these hematological parameters at stroke onset in our study might support underlying complex pathophysiology and further investigation is needed.

Our study supports a better correlation between reduced on-treatment platelet activity, indicated by prolonged CEPI-CT in 2nd measurement with favorable outcome in the Pre-ASA group compared to Post-ASA group. However, the generalizability of using only PFA-100 measurements taken prior to aspirin might be difficult in real-world practice since aspirin is commonly prescribed and available over the counter. Our results demonstrated that platelet function measurements during the acute stages of stroke, even after aspirin treatment, correlate with late functional outcomes.

Based on our study findings, we recommend that patients with acute ischemic stroke having the initial PFA-100 measurement as soon as possible at onset, regardless of prior aspirin treatment, followed by a second measure 2 weeks later. If the results are not sufficiently prolonged at the second measurement, it is advisable to thoroughly assess patient compliance, review possible medication interactions, and more strict control of risk factors. Options such as increasing the dose of aspirin, re-examining the PFA-100, or adding or shifting to another antiplatelet agent may be considered. However, there is currently no strong evidence to support adjustments to antiplatelet therapy.

This study has several limitations. Not all baseline PFA-100 measurements at the onset of acute cerebral infarction were performed before taking aspirin. In real world practice, the priority was to provide participants with the earliest antiplatelet effects, followed by study compliance with the protocol requirements. Additionally, the small sample sizes of the Pre-ASA and Post-ASA subgroups limit the statistical power of our findings. As a result, the conclusions drawn from these subgroup analyses should be interpreted with caution. Larger, multi-center studies are needed to validate these findings and assess their generalizability to broader populations. The compliance of AIS participants with aspirin intake could not be completely ascertained after discharge.

Some disadvantages of PFA-100 included inconsistent reference ranges [2], dependence on von Willebrand factor (vWF) levels [48], hematocrits, platelet counts, and insensitivity to clopidogrel [49,50]. The clinical practice has been becoming more complex, as increasing evidence now supports dual antiplatelet therapy (DAPT) with aspirin and clopidogrel in high-risk TIA [51] and minor ischemic stroke patients [52]. Moreover, DAPT with aspirin and ticagrelor has even better outcomes in patients with CYP2C19 loss of function (LOF) alleles compared to aspirin and clopidogrel [53]. Given that over 50% of Asian population carries CYP2C19 LOF alleles, there is likely no simple diagnostic or treatment pathway for monitoring antiplatelet use in these patients. This might include CYP2C19 genotyping, platelet function tests sensitive to clopidogrel, and further well-designed clinical studies.

In conclusion, our study demonstrated that changes in serial platelet function measurement taken at the presentation of acute stroke, whether before or after aspirin treatment, and 2 weeks later under medication might predict clinical outcomes, a finding not previously reported in studies relying solely on on-treatment cutoff measurements. The generalizability of our study results might be further ascertained through additional investigations, emphasizing on a larger study population, prespecified timing of platelet function tests before and after antiplatelet therapy, and follow-up length in the context of the study design after AIS.

## Figures and Tables

**Table 1:** Demographic Characteristics of the Participants.

Characteristics	Cerebral Infarct (*n* = 212)	Health Control (*n* = 112)	*p* Value
Age, years; mean ± SD	65.05 ± 14.42	51.70 ± 9.27	<0.001 *
Sex, *n* (%)			0.001
Male	135 (63.68)	50 (44.64)	
From stroke onset to screening, days; mean ± SD	2.32 ± 5.76		
Main predictor (Baseline) mean ± SD			
Platelet closure time, EPI (s)	181.71 ± 81.50	137.63 ± 57.50	<0.001 *
<82, *n* (%)	10 (4.72)	3 (2.68)	
82–150	84 (39.6)	82 (73.2)	
151–250	58 (27.4)	15 (13.4)	
>250	60 (28.3)	12 (10.7)	
Platelet closure time, ADP (s)	89.45 ± 36.56	87.03 ± 22.03	0.4584
<62, *n* (%)	27 (12.74)	6 (5.36)	
62–100	132 (62.26)	77 (68.75)	
>100	53 (25.00)	29 (25.89)	
Baseline PFA measurement between received antiplatelet drug ^1^, mean ± SD			
	Cerebral Infarct		
	Pre-ASA	Post-ASA	
*n* (%)	86 (40.95%)	124 (59.05%)	
Platelet closure time, EPI (s)	141.0 ± 68.65	209.82 ± 78.29	<0.001 *
Platelet closure time, ADP (s)	83.41 ± 31.44	93.36 ± 39.54	0.044 *

^1^ Two subjects were transferred from other hospitals to our hospital, making it impossible to determine whether they had taken anticoagulant/antiplatelet medications at other hospitals; therefore, they were excluded. * Significant different results. SD, standard deviation; IQR, interquartile range; s, seconds; NIHSS, National Institutes of Health Stroke Scale; mRS, modified Rankin Scale; BI, Barthel index; PTT, Partial thromboplastin time; INR, international normalizing ratio; EPI, Epinephrine; and ADP, Adenosine diphosphate; Pre-ASA, pre-aspirin; Post-ASA, post-aspirin.

**Table 2:** Characteristics of Ischemic Stroke Participants Classified According to Favorable (mRS 0–3) or Unfavorable Outcome.

Characteristics	Baseline (*n* = 212)	One Month Follow-Up			One Year Follow-Up		
		Favorable (*n* = 158)	Unfavorable (*n* = 54)	*p* Value	Favorable (*n* = 164)	Unfavorable (*n* = 48)	*p* Value
Age, years, mean ± SD	65.05 ± 14.42	62.27 ± 13.59	73.20 ± 13.78	<0.001 *	62.02 ± 13.36	75.42 ± 13.16	<0.001 *
Sex, *n* (%)				0.0007 *			0.004 *
Male	135 (63.68)	111 (70.25)	24 (44.44)		113 (68.90)	22 (45.83)	
Female	77 (36.32)	47 (29.75)	30 (55.56)		51 (31.10)	26 (54.17)	
Medical history, *n* (%)							
Hypertension ^1^	159 (75.0)	115 (72.78)	44 (81.48)	0.203	119 (72.56)	40 (83.33)	0.130
Diabetes mellitus ^1^	80 (37.74)	55 (34.81)	25 (46.30)	0.133	65 (39.63)	15 (31.25)	0.292
Dyslipidemia ^1^	102 (48.11)	78 (49.37)	24 (44.44)	0.532	83 (50.61)	19 (39.58)	0.179
Uremia ^1^	3 (1.42)	1 (0.63)	2 (3.70)	0.160	1 (0.61)	2 (4.17)	0.129
Atrial fibrillation ^1^	10 (4.72)	5 (3.16)	5 (9.26)	0.079	6 (3.66)	4 (8.33)	0.167
Previous CVA ^1^	40 (18.87)	24 (15.19)	16 (29.63)	0.019 *	24 (14.63)	16 (33.33)	0.004 *
Smoker ^1^	76 (35.85)	62 (39.24)	14 (25.93)	0.078	67 (40.85)	9 (18.75)	0.005 *
Laboratory values, mean ± SD							
Hemoglobin (g/dL)	14.06 ± 1.74	14.32 ± 1.70	13.30 ± 1.66	0.0002 *	14.31 ± 1.68	13.19 ± 1.67	<0.001 *
Hematocrit (%)	44.22 ± 25.35	43.34 ± 9.25	46.76 ± 47.91	0.604	43.30 ± 9.10	47.34 ± 50.83	0.586
White Blood Cells (×10^9^/L)	9.52 ± 7.48	9.68 ± 8.45	9.07 ± 3.35	0.454	8.90 ± 5.33	11.65 ± 12.13	0.132
Platelet count (×10^9^/L)	235.25 ± 77.97	236.96 ± 85.90	230.22 ± 48.05	0.477	238.67 ± 85.40	223.54 ± 42.61	0.097
Neutrophil count (×10^9^/L)	6.46 ± 5.44	6.46 ± 5.98	6.45 ± 3.48	0.983	5.98 ± 3.91	8.06 ± 8.75	0.119
Lymphocyte count (×10^9^/L)	2.47 ± 3.15	2.64 ± 3.60	1.98 ± 0.97	0.034 *	2.35 ± 1.65	2.89 ± 5.90	0.539
Monocyte count (×10^9^/L)	0.47 ± 0.38	0.47 ± 0.41	0.47 ± 0.29	0.989	0.45 ± 0.33	0.56 ± 0.52	0.165
Eosinophil count (×10^9^/L)	0.16 ± 0.18	0.15 ± 0.18	0.17 ± 0.19	0.537	0.16 ± 0.18	0.16 ± 0.20	0.789
Basophil count (×10^9^/L)	0.03 ± 0.03	0.03 ± 0.03	0.03 ± 0.02	0.691	0.03 ± 0.03	0.03 ± 0.05	0.566
Glucose (mg/dL)	169.09 ± 90.45	166.8 ± 90.75	176.2 ± 90.04	0.516	169.5 ± 90.66	167.8 ± 90.71	0.909
NLR	3.94 ± 6.86	3.33 ± 3.05	5.7 ± 12.45	0.176	3.51 ± 2.69	6.62 ± 13.28	0.081
PLR	129.68 ± 75.24	122.8 ± 66.08	149.7 ± 95.04	0.061	121.7 ± 62.59	156.7 ± 104.1	0.032 *
LMR	5.98 ± 5.84	6.27 ± 6.51	5.16 ± 3.10	0.101	5.93 ± 2.55	6.16 ± 11.42	0.895
PTT (s)	28.18 ± 3.68	28.22 ± 2.99	28.06 ± 5.43	0.880	28.28 ± 2.93	27.72 ± 6.17	0.687
INR	27.52 ± 3.54	27.42 ± 3.18	27.85 ± 4.54	0.638	27.44 ± 3.33	27.90 ± 4.44	0.589
Baseline scores of clinical outcomes							
NIHSS (mean ± SD)	5.73 ± 6.98	3.32 ± 3.38	12.76 ± 9.61	<0.001 *	3.70 ± 3.77	12.67 ± 10.28	<0.001 *
≤8, *n* (%)	171 (80.66)	150 (94.94)	21 (38.89)	<0.001 *	151 (92.07)	20 (41.67)	<0.001 *
>8	41 (19.34)	8 (5.06)	33 (61.11)		13 (7.93)	28 (58.33)	
GCS total score, median (IQR)	15.0 (15.0–15.0)	15.0 (15.0–15.0)	14.0 (10.0–15.0)	<0.001 *	15.0 (15.0–15.0)	14.0 (10.0–15.0)	<0.001 *
Barthel index, mean ± SD	77.50 ± 33.42	94.57 ± 13.44	26.60 ± 20.98	<0.001 *	90.90 ± 19.49	30.74 ± 29.67	<0.001 *

^1^ Past history was considered. * Significant different results. s, seconds; SD, standard deviation; IQR, interquartile range; mRS, modified Rankin Scale; CVA, cerebrovascular accident; PTT, Partial thromboplastin time; INR, international normalizing ratio; NIHSS, National Institutes of Health Stroke Scale; NLR, Neutrophil-to-Lymphocyte ratio; PLR, Platelet-to-Lymphocyte ratio; LMR, Lymphocyte-to-Monocyte ratio.

**Table 3:** Differences in Sequential PFA-100 Measurements Between Participants with Favorable (mRS 0–3) or Unfavorable Outcomes.

Characteristics	One Month Follow-Up			One Year Follow-Up		
	Favorable (*n* = 158)	Unfavorable (*n* = 54)	*p* Value	Favorable (*n* = 164)	Unfavorable (*n* = 48)	*p* Value
Onset to 1st PFA (days)	1.93 ± 4.92	3.44 ± 7.67	0.179	1.98 ± 4.81	3.48 ± 8.20	0.231
1st PFA to 2nd PFA (days)	16.57 ± 24.68	14.26 ± 21.96	0.542	16.91 ± 25.01	12.79 ± 19.99	0.296
1st CEPI-CT (s), mean ± SD	180.32 ± 80.76	185.80 ± 84.26	0.671	178.99 ± 80.91	191.02 ± 83.69	0.369
2nd CEPI-CT (s), mean ± SD	220.97 ± 81.72	169.62 ± 77.74	0.0003 *	215.26 ± 82.18	180.83 ± 83.59	0.019 *
2nd > 150 (s), *n* (%)	91 (57.59)	20 (37.04)	0.115	90 (54.88)	21 (43.75)	0.344
2nd > 193	78 (49.37)	14 (25.93)	0.784	76 (46.34)	16 (33.33)	0.734
3rd CEPI-CT (s), mean ± SD	203.80 ± 81.49	166.60 ± 78.12	0.012 *	202.34 ± 82.24	167.50 ± 76.02	0.023 *
Difference by CEPI-CT (s), mean ± SD						
1st and 2nd	42.12 ± 101.23	−15.91 ± 100.69	0.0009 *	38.98 ± 101.20	−12.71 ± 104.36	0.005 *
1st and 3rd	23.08 ± 104.31	−11.08 ± 104.40	0.071	23.71 ± 105.38	−17.31 ± 98.35	0.036 *
2nd and 3rd	−14.90 ± 99.04	5.94 ± 103.32	0.297	−9.57 ± 98.46	−11.50 ± 107.55	0.926

PFA, platelet function analysis; CEPI, collagen and epinephrine; CT, closure time; s, seconds; 1st, day 1 measurement; 2nd, week 2 measurement; 3th, week 4 measurement; * Significant different results.

**Table 4:** Multivariable Logistic Regression Analysis of the Difference in PFA-100 and Favorable (mRS 0–3) vs. Unfavorable Outcomes.

	One Month Follow-Up (*n* = 177)			One Year Follow-Up (*n* = 177)		
	OR (95% CI)	AUC (95% CI)	*p* Value	OR (95% CI)	AUC (95% CI)	*p* Value
Change from baseline to week 2 CEPI-CT						
Model 1	1.006 (1.002–1.009)	0.67 (0.58–0.76)	0.002 *	1.005 (1.001–1.008)	0.66 (0.56–0.75)	0.006 *
Model 2	1.007 (1.003–1.011)	0.79 (0.72–0.87)	0.001 *	1.006 (1.002–1.010)	0.81 (0.74–0.88)	0.006 *
Model 3	1.007 (1.002–1.012)	0.91 (0.86–0.96)	0.008 *	1.005 (1.000–1.010)	0.90 (0.85–0.95)	0.040 *
Model 4	1.008 (1.002–1.013)	0.93 (0.88–0.97)	0.005 *	1.006 (1.001–1.011)	0.91 (0.85–0.97)	0.021 *
Q1 (−218 to −23)	reference	--	--	reference	--	--
Q2 (−23 to 21)	3.873 (0.953–15.740)		0.058	7.380 (1.708–31.889)		0.007 *
Q3 (21 to 99)	3.213 (0.795–12.989)		0.102	3.546 (0.836–15.037)		0.086
Q4 (99 to 246)	11.859 (2.318–60.669)		0.003 *	9.807 (2.078–46.284)		0.004 *
Change from baseline to week 4 CEPI-CT						
Model 4	1.004 (1.00–1.008)	0.86 (0.79–0.93)	0.083	1.005 (1.00–1.009)	0.88 (0.82–0.95)	0.049 *
Change from week 2 to week 4 CEPI-CT						
Model 4	0.996 (0.991–1.001)	0.88 (0.81–0.95)	0.157	0.999 (0.994–1.005)	0.89 (0.82–0.97)	0.854

PFA, platelet function analysis; CEPI, collagen and epinephrine; CT, closure time; OR, odd ratio; 95% CI, confidence intervals; Q1, The first quartile; Q2, The second quartile; Q3, The third quartile; Q4, The fourth quartile. Model 1, non-adjusted model; Model 2, adjusted for age and sex; Model 3, adjusted for age, sex and subgroup-NIHSS (<8 vs. ≥8); Model 4, adjusted for age, sex and subgroup-NIHSS (<8 vs. ≥8), previous CVA, hemoglobin, neutrophil-to-lymphocyte ratio, platelet-to-lymphocyte ratio; * Significant different results.

## Data Availability

The datasets generated during and/or analyzed during the current study are available from the corresponding author on reasonable request.

## Supplementary Materials

The following supporting information can be downloaded at: https://www.mdpi.com/article/10.3390/diagnostics14242848/s1, Table S1. Sequential PFA-100 Measurements in Pre-ASA subgroup: Relationship with Favorable (mRS 0–3) and Unfavorable Outcomes. Table S2. Sequential PFA-100 Measurements in Post-ASA subgroup: Relationship with Favorable (mRS 0–3) and Unfavorable Outcomes. Table S3. Multivariable Logistic Regression Analysis of Association Between CEPI-CT Change from Baseline to Week 2 and Favorable (mRS 0–3) vs. Unfavorable Outcomes.
