# Supplementary material for: Serial Examination of Platelet Function Tests Might Predict Prognosis of Patients with Acute Ischemic Stroke—A Cohort Study
Source: Diagnostics (Basel). 2024 Dec 18;14(24):2848. doi: 10.3390/diagnostics14242848 (PMC11675241; doi:10.3390/diagnostics14242848)
Supplement: Supplementary file 1 [file diagnostics-14-02848-s001.zip › diagnostics-3294748-supplementary.pdf]

Supplement Table S1. Sequential PFA-100 Measurements in **Pre-ASA** subgroup: Relationship with Favorable (mRS 0–3) and Unfavorable Outcomes

	**1-month** follow-up			**1-year** follow-up		
Characteristics	Favorable (n=67)	Unfavorable (n=19)	*p* value	Favorable (n=68)	Unfavorable (n=18)	*p* value
Onset to 1st PFA (days), mean±SD	0.18±6.17	1.37±2.97	0.246	0.28±6.07	1.06±3.57	0.606
1st PFA to 2nd PFA (days), mean±SD	11.31±8.83	13.37±19.58	0.661	12.54±13.27	8.83±2.43	0.003*
1st CEPI-CT (sec), mean±SD	138.7±67.85	149.3±72.69	0.555	136.1±66.54	159.6±75.19	0.199
2nd CEPI-CT (sec), mean±SD	222.9±78.51	161.3±65.23	0.003*	213.9±79.49	189.2±79.55	0.262
3rd CEPI-CT (sec), mean±SD	204.4±79.04	151.7±86.41	0.031*	201.4±81.49	161.5±82.50	0.115
Difference by CEPI-CT (seconds), mean±SD						
1st and 2nd	87.92±94.90	9.0±81.86	0.002*	81.37±95.68	27.47±94.74	0.043*
1st and 3rd	61.15±107.5	15.50±94.51	0.148	63.52±108.5	0.85±77.41	0.052
2nd and 3rd	-24.75±95.82	3.54±112.5	0.361	-15.55±96.28	-34.75±114.0	0.549

Pre-ASA subgroup: baseline PFA-100 measurement was performed before receiving aspirin

PFA, platelet function analysis; CEPI, collagen and epinephrine; CT, closure time; sec, seconds; 1st, day 1 measurement; 2nd, week 2 measurement; 3th, week 4 measurement; * Significant different results.

Supplement Table S2. Sequential PFA-100 Measurements in **Post-ASA** subgroup: Relationship with Favorable (mRS 0–3) and Unfavorable Outcomes

	**1-month** follow-up			**1-year** follow-up		
Characteristics	Favorable (n=89)	Unfavorable (n=35)	*p* value	Favorable (n=94)	Unfavorable (n=30)	*p* value
Onset to 1st PFA (days)	3.16±3.20	4.57±9.13	0.377	3.12±3.18	4.93±9.78	0.325
1st PFA to 2nd PFA (days)	19.17±28.55	14.74±23.41	0.417	18.80±27.89	15.17±25.07	0.526
1st CEPI-CT (sec), mean±SD	211.5±76.20	205.6±84.37	0.710	209.8±76.86	209.9±83.99	0.995
2nd CEPI-CT (sec), mean±SD	218.4±85.34	174.8±85.29	0.023*	215.9±85.27	175.1±87.37	0.043*
3rd CEPI-CT (sec), mean±SD	205.4±83.90	174.6±73.81	0.102	205.3±83.29	170.9±73.81	0.078
Difference by CEPI-CT (seconds), mean±SD						
1st and 2nd	4.09±91.12	-31.38±109.3	0.100	5.09±93.49	-40.04±103.4	0.045*
1st and 3rd	-5.66±91.62	-25.38±108.4	0.372	-5.78±92.17	-27.57±108.7	0.343
2nd and 3rd	-3.17±101.3	7.5±99.90	0.688	-1.66±100.3	4.0±103.4	0.837

Post-ASA subgroup: baseline PFA-100 measurement was performed after receiving aspirin

PFA, platelet function analysis; CEPI, collagen and epinephrine; CT, closure time; sec, seconds; 1st, day 1 measurement; 2nd, week 2 measurement; 3th, week 4 measurement; * Significant different results.

Supplement Table S3. Multivariable Logistic Regression Analysis of Association Between CEPI-CT Change from Baseline to Week 2 and Favorable (mRS 0–3) vs. Unfavorable Outcomes

	**1-month** follow-up			**1-year** follow-up		
**Pre-ASA** (n=86)	OR (95% CI)	AUC (95% CI)	*p* value	OR (95% CI)	AUC (95% CI)	*p* value
Model 1	1.009 (1.003-1.015)	0.75 (0.63-0.87)	0.005*	1.006 (1.000-1.012)	0.66 (0.51-0.81)	0.050*
Model 2	1.013 (1.005-1.021)	0.78 (0.68-0.92)	0.001*	1.010 (1.002-1.017)	0.84 (0.75-0.93)	0.012*
Model 3	1.011 (1.002-1.019)	0.86 (0.75-0.96)	0.011*	1.007 (0.999-1.015)	0.87 (0.78-0.95)	0.096
Model 4	1.012 (1.003-1.021)	0.88 (0.78-0.97)	0.007*	1.008 (0.999-1.016)	0.88 (0.80-0.96)	0.077
	**1-month** follow-up			**1-year** follow-up		
**Post-ASA** (n=124)	OR (95% CI)	AUC (95% CI)	*p* value	OR (95% CI)	AUC (95% CI)	*p* value
Model 1	1.004 (0.999-1.008)	0.66 (0.50-0.76)	0.103	1.005 (1.000-1.010)	0.66(0.53-0.79)	0.049*
Model 2	1.003 (0.998-1.009)	0.80 (0.71-0.89)	0.209	1.004 (0.999-1.010)	0.81 (0.71-0.91)	0.130
Model 3	1.008 (0.999-1.018)	0.96 (0.93-0.99)	0.081	1.008 (1.001-1.016)	0.94 (0.88-0.99)	0.034*
Model 4	1.010 (0.999-1.021)	0.97 (0.95-1.00)	0.065	1.010 (1.002-1.019)	0.95 (0.90-1.00)	0.021*

Pre-ASA: subgroup with baseline PFA-100 measurement was performed before receiving aspirin

Post-ASA: subgroup with baseline PFA-100 measurement was performed after receiving aspirin

PFA, platelet function analysis; CEPI, collagen and epinephrine; CT, closure time; OR, odd ratio; 95% CI, confidence intervals

Model 1, non-adjusted model; Model 2, adjusted for age and sex; Model 3, adjusted for age, sex and subgroup-NIHSS (<8 vs. ≥8); Model 4, adjusted for age, sex and subgroup-NIHSS (<8 vs. ≥8), previous CVA, hemoglobin, neutrophil-to-lymphocyte ratio, platelet-to-lymphocyte ratio; * Significant different results.
